# One Step Fabrication of Highly Absorptive and Surface Enhanced Raman Scattering (SERS) Silver Nano-trees on Silicon Substrate

**DOI:** 10.1038/s41598-019-49896-2

**Published:** 2019-09-19

**Authors:** Sara Abdel Razek, Ahmed B. Ayoub, Mohamed A. Swillam

**Affiliations:** Department of Physics, School of Sciences and Engineering, American University of Cairo, New Cairo, 11835 Egypt

**Keywords:** Synthesis and processing, Nanowires, Synthesis and processing, Nanowires, Synthesis and processing

## Abstract

Silver Nano-trees (AgNTs) were synthesized by one-step electroless method with different densities via water or ethylene glycol (EG) on silicon substrate in one minute. The density of AgNTs is controlled by changing the concentration of silver nitrate in etchant solution. The absorption of NTs fabricated via EG is higher than absorption of NTs without EG. The AgNTs are employed as substrates for surface-enhanced Raman scattering (SERS) and exhibit high sensitivity. The silver Nano-trees fabricated via ethylene glycol (AgNTs-EG) enhances the Raman spectrum of pyridine (Py) with higher enhancement factor. Moreover, the SERS-active substrates prepared by using EG were able to detect Pyridine with concentration as low as 0.005 mM, the ones fabricated by water could only detect Pyridine at concentration of 0.2 mM.

## Introduction

Recently, nanostructures of noble metals have been quickly developed into promising applications such as catalysis, electronic systems, sensing, and surface enhanced Raman scattering (SERS)^1,2^ due to their unique electrical, optical and catalytic properties that are different from the bulk metals. The metal nanostructures have intrinsic properties that can be tuned by controlling their size, shape and crystallinity as shown in^3–8^. SERS is sensitive to the interface with metal nanoparticles^9,10^. The size and shape of the nanoparticles have strong effect on the strength of the Raman enhancement^11^. Nano-particles optimum size is wavelength-dependent, and it is in a range from about 50 to 100 nm from the visible to the NIR range^12^. The metal Tree-like nanostructure is appropriate for enhancing the SERS effect^13^. One of the enhancement mechanisms which is known as electromagnetic enhancement of the silver film is correlated with the excitation of surface plasmons through the Nano-trees. Excited surface plasmons on the tip of Nano-trees are associated with collective electron oscillations, which create a localized electro- Magnetic field (EM). Another type of the enhancement mechanisms (denoted as chemical enhancement) is correlated with the chemical adsorption absorption of Pyridine (Py) on silver^14^.

Among different metallic nanostructures, silver nanostructures have gained interest in different applications due to its low toxicity. It supports surface plasmons (SPs) in the visible and near-infrared regions (NIR), which leads to fabrication of more effective SERS active substrates^15,16^. Their UV/VIS spectrum, known as the surface plasmon absorption band (SPAB), is generated by the oscillation of the conduction electrons in the particles as a result of the incident light. The SPAB is affected by the size^17–19^, shape^20^, poly-dispersity^21^, aggregation^22,23^, surrounding medium, and geometric arrangements of metal nanoparticles^21^. Silver also has the highest thermal and electrical conductivities among all metals^24,25^. Therefore, silver nanoparticles have found great areas of applicability owing to their optical, thermal, and electrical properties. One of the most common nanostructures is silver dendrites that called silver Nano-trees (AgNTs), which consist of different generations of branches^26–29^. Multi-levels of branching structures increase the specific surface area of substrate. AgNTs may support EM coupling in the space between two adjacent branches due to coupling of the surface plasmon polaritons (SPP). Consequently, the generation of “hot spots” increases in the spacing at the end and among branches of silver. Although different silver nanostructures have high SERS properties, the surfaces are extremely sensitive to oxidation; accordingly, the time stability in SERS application is low.

Anisotropic crystal growth and diffusion-limited aggregation (DLA) effect plays important roles in the fabrication of Nano-trees^30–35^. As a result, the variety of the dendritic structures can be realized by balancing these two factors. There are many theoretical attempts to explain the process of growing dendrites^36^. Ref.^36^ reported that the anisotropy of the solid–liquid interfacial energy, g (n~) determines the orientation selection. An oriented attachment mechanism was improved to explain the variety of hierarchical dendrite 0. The competition between the kinetic and thermodynamic factors is responsible for converting the fractal pattern to Ag Nano-trees.

Ethylene glycol (EG) can be utilized as reducing agent to synthesize metal nanoparticles by the polyol process^37^. EG can be used for producing silver nanoparticles as solvent and reducing agent to increase the stability of the obtained Ag colloid without adding costabilizers. Moreover, the semiconducting substrate such as Si can be used for enhancing the effect of the SERS and improving the long-term stability by preventing the oxidization of silver^38,39^.

In this paper, we propose a simple one-minute electroless etching process to fabricate AgNTs on Si substrate by using two types of etchant solutions with different concentrations of silver nitrate at room temperature. The AgNTs were fabricated using a large-sized Si substrate to increase ability of detection of pyridine. High absorption is achieved using etchant solution. The optical properties of NTs have been investigated.

## Results and Discussion

**Figure 1:**
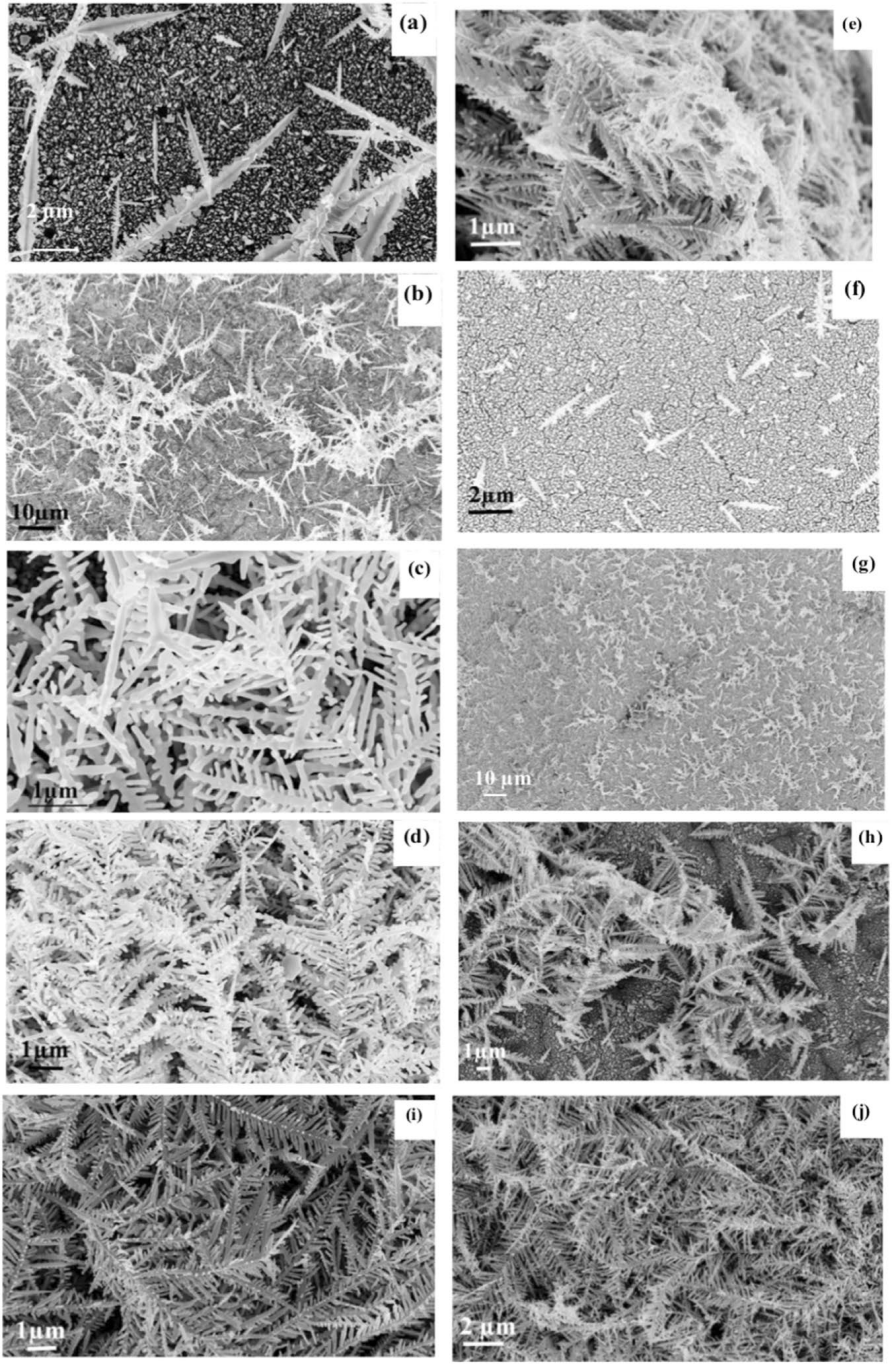
FESEM of the fabricated Ag Nano-trees in two different solutions: group A (without ethylene glycol) when the concentration of silver nitrate was (**a**) 5 mM, (**b**) 7.5 mM, (**c**) 10 mM, (**d**) 12.5 mM, and (**e**) 15 mM, group B (with ethylene glycol) when the concentration of silver nitrate was (**f**) 5 mM, (**g**) 7.5 mM, (**h**) 10 mM, (**i**) 12.5 mM, and (**j**) 15 mM.

**Figure 2:**
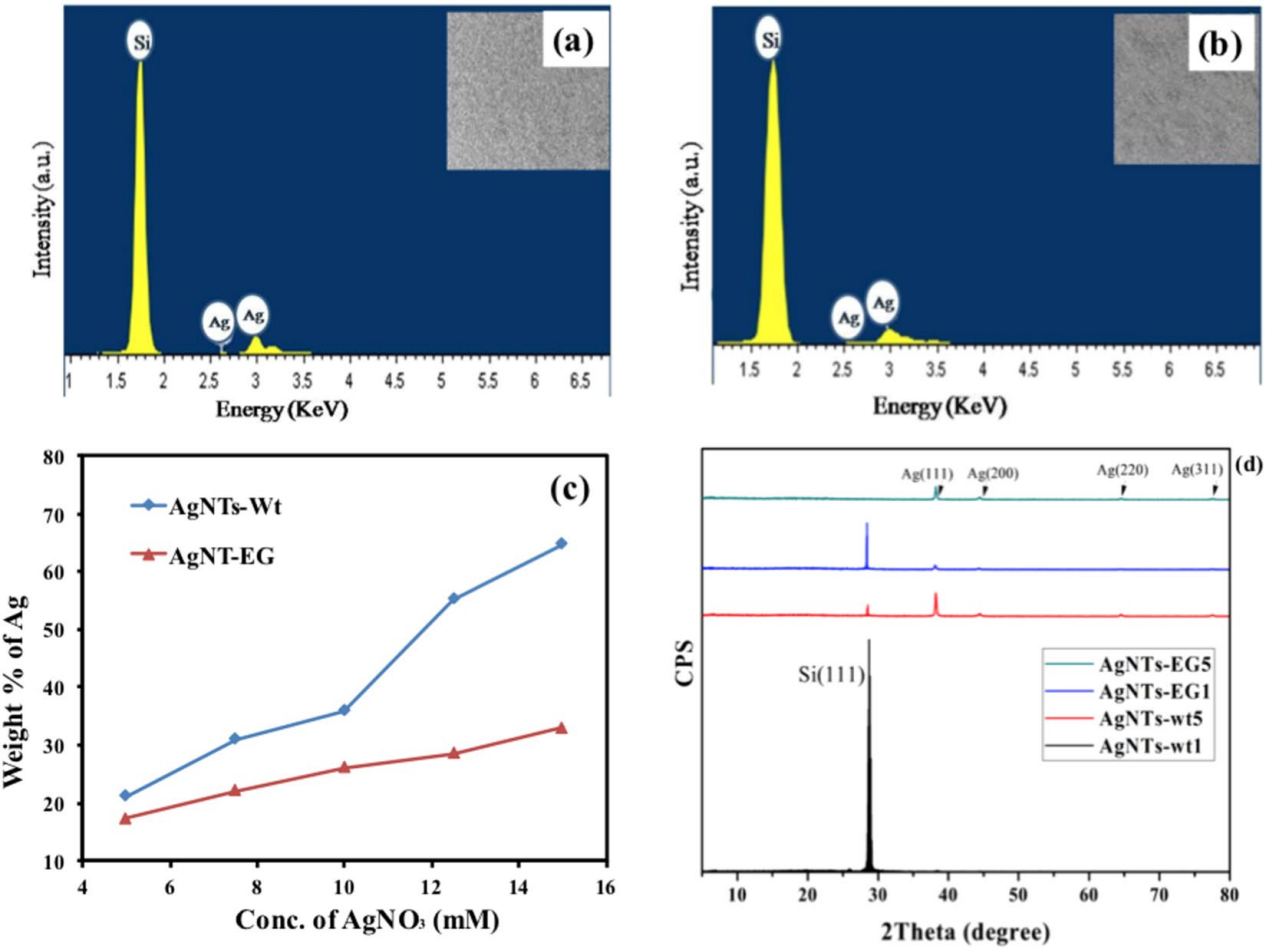
EDX spectra of (**a**) AgNT-wt1, (**b**) AgNT-EG1, and (**c**) relationship between weight % of Ag and concentration of AgNO_3_ for two types of fabricated NTs, and (**d**) XRD patterns of AgNTs concentration. Although the (AgNTs-wt5) and the (AgNTs-EG5) samples experience Ag peaks in different orientation, it could be observed from the figure that the peak along the (111) axis is the dominating one from the XRD patterns. Hence, the long central branches obviously point to the preferred growth direction along the (111) axis. Each Nano-tree has a sharp secondary branch that has few secondary branches.

Figure 1 shows the SEM images of the obtained AgNTs by using the two different aqueous solutions. The morphology of the fabricated AgNTs was examined using a Zeiss Lro Supra 55 field emission scanning electron microscope (FESEM) to show the effect of using ethylene glycol in the etchant solution. The growth speed of AgNTs became even faster with the increase of concentration of silver nitrate, because AgNT can capture silver ions more rapidly in shorter mean free path through the growth process and reduce it into silver^38^. The number of the fabricated AgNTs increases with increasing the concentration of silver nitrate in the two solutions and have long central backbone. The existence of ethylene glycol in second solution generates colloidal Nano-trees. The energy dispersive x-ray (EDX) spectra of samples in two groups have been measured and shown in Fig. 2a, b to confirm the existence of silver. As shown in Fig. 2, it indicates the presence of Ag and Si substrate. Figure 2c shows the relationship between the concentration of silver nitrate and the weight percentage of silver deposited on silicon for two groups. The weight percentage of silver deposited on silicon when the solution contained water only, is higher than the weight percentage of silver deposited on silicon in existence of ethylene glycol in all concentrations of AgNO_3_. Figure 2d shows the typical X-ray diffraction (XRD) patterns of fabricated AgNTs by two different concentrations of silver nitrate (5, 15 mM) in two solutions. The XRD analysis was performed using Cu K*α*1 radiation source (*λ* = 1.5406 A°). The XRD patterns show sharp peaks that are indexed to (111), (200), (220), (311) diffraction peaks of the FCC Ag, and Si (111) diffraction peak comes from the Si substrate. The intensities of silicon peak of the first (AgNTs-wt1) and third (AgNTs-EG1) samples were higher than the second (AgNTs-wt5) and fourth (AgNTs-EG5) samples due to the low concentration of deposited silver. Moreover, the intensities of Ag peaks with all orientations, of the second (AgNTs-wt5) and fourth (AgNTs-EG5) samples were greater than the first (AgNTs-wt1) and third (AgNTs-EG1) samples due to increasing of silver.

## Discussion

### Optical properties

**Figure 3:**
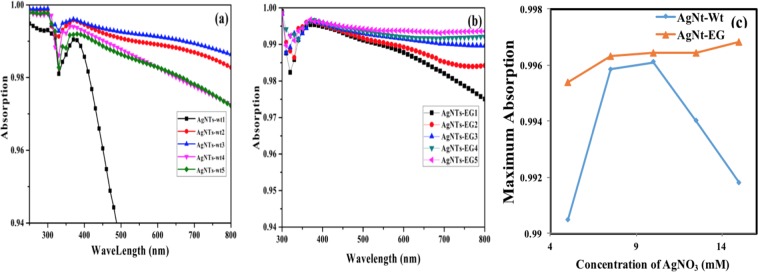
Absorption of fabricated AgNTs with different concentrations of AgNO_3_ (**a**) when the etchant solution contained water only, (**b**) when the etchant solution contained ethylene glycol and (**c**) relationship between maximum absorption of all fabricated NTs and concentration of AgNO_3_ at wavelength 370 nm (due to maximum absorption at this wavelength).

The absorption spectra as shown in (Fig. 3) was measured using a Perkin-Elmer-Lambda UV–NIR and visible spectrophotometer with solid-sample holder for reflectance measurements and a universal reflectance unit where the reflection “R” is measured and then the absorption is measured as “A = 1 − R”. The absorption of AgNTs fabricated without ethylene glycol increases with increasing the concentration of AgNO_3_ in the first three concentrations, then the absorption decreases with continuous increase of AgNO_3_ due to increase of density of the fabricated AgNTs. The increased density of AgNTs enhances the scattering of light in the last two samples.

On the other hand, the absorption of the AgNTs synthesized in the presence of ethylene glycol increases with increasing the concentration of silver nitrate. The ethylene glycol here worked as reducing agent to increase the stability of the obtained silver collides. Generally, the absorption of silicon, throughout the entire wavelength range, increased by adding AgNTs of different densities due to the multiple reflection of light through AgNTs, i.e. trapping light. The absorption of the AgNTs-EG is larger than absorption of AgNTs-wt in all various concentrations of AgNO_3_ as shown in Fig. 3 due to the high refractive index of ethylene glycol^39^. In Ref.^40^, the same study was performed for silver nanoparticles (AgNPs). The AgNPs experienced higher absorption when immersed in ethylene glycol not water, since the higher refractive index of the ethylene resulted in higher extinction cross section (an indication on absorption)^41^ through Eq. (1)

1 $${C}_{ext}=9\,[\frac{\omega }{c}{\varepsilon }_{m}^{3/2}\frac{{\varepsilon }_{i}}{{({\varepsilon }_{r}+2{\varepsilon }_{m})}^{2}+{\varepsilon }_{i}^{2}}]$$

where ${\varepsilon }_{m}$ is the permittivity of the surrounding medium, ${\varepsilon }_{i}$ is the imaginary part of the metal permittivity, and ${\varepsilon }_{r}$ is the real part of the metal permittivity. The increase in the extinction cross section results in higher absorption coefficient. Although AgNTs differ from AgNPs in their geometrical structure, however, the general rules of scattering and absorption still hold and similar trends in the absorption properties are observed with different values. For example, when the silver nitrate concentration was 5 mM, the maximum absorption of AgNTs-wt1 was 0.9904 compared with AgNTs-EG1 that was 0.995 at wavelength 370 nm, which is a small difference 0.0046 as shown in Fig. 3a. The difference between the two absorption patterns increased to 0.13434 at wavelength of 800 nm. The absorption increasing in AgNTs-wt was continued till the concentration of AgNO_3_ was 10 mM to reach its maximum absorption 0.9961 at 370 nm, then started to decrease to reach 0.9918 for 15 mM of AgNO_3_. In contrast, the absorption of AgNTs-EG continued to increase with increasing the AgNO_3_ concentration to reach its maximum *≈* 0.997 for 15 mM of AgNO_3_ at wavelength 370 nm. Moreover, the absorption of AgNTs-EG5 was larger than absorption of AgNTs-wt5 by (*≈* 0.005) in wavelength 370 nm and the difference was increased in higher wavelength to be 0.0213 at wavelength of 800 nm as shown in Fig. 3b. The increasing and decreasing in absorbance of AgNTs fabricated without EG depends on the density of the fabricated AgNTs. When the density of the AgNTs was small, the absorption is enhanced by increasing the trapping of light through the trees till light trapping process is transformed into light scattering in high densities of Ag. Figure 3c presents a maximum absorption of silver Nano-trees fabricated in water and ethylene glycol solutions as a function of AgNO_3_ concentration. Moreover, there is a small red shift in wavelength of absorption of AgNTs-EG that is around 10 nm due to high refractive index of ethylene glycol^39^. This shift is due to the resonance peak shift in the polarizability. Referring to NPs^41^, polarizability is given by

2 $$\alpha =4\pi {a}^{3}\frac{\varepsilon (\omega )-{\varepsilon }_{m}}{{(\varepsilon (\omega )+2{\varepsilon }_{m})}^{2}}$$

From Eq. (2), the polarizability is inversely proportional with ${(\varepsilon (\omega )+2{\varepsilon }_{m})}^{2}$, where $\varepsilon (\omega )\,\,$is the permittivity of the metal and ${\varepsilon }_{m}$ is the permittivity of the surrounding medium. From this relation, it is obvious that the higher medium permittivity leads to resonance at longer wavelength, and hence the absorption resonant wavelength experiences a red shift in wavelength. Therefore, ethylene glycol results in a red shift in the absorption resonant wavelength as compared to water owing to its higher refractive index.

### Raman spectroscopy

Raman spectroscopy provides information on vibrational, molecular, and electronic levels. Raman scattering is one of the most powerful techniques utilized to identify chemical and biological samples through the detection of the characteristic oscillations of the molecules contained in samples. Through this phenomenon, molecules adsorbed onto a metal surface under specific conditions show a large interaction cross-section for the Raman effect, as a result, the Raman signal of these molecules is amplified by several orders of magnitude^42–44^. To find out the detection limit for pyridine (Py) on AgNTs fabricated on silicon substrate by electroless method, we measured the Raman spectra of Py applied to 8 samples. Pyridine was chosen as a test compound for investigating the application of AgNT structure to detect the trace of toxic organic compounds.

**Figure 4:**
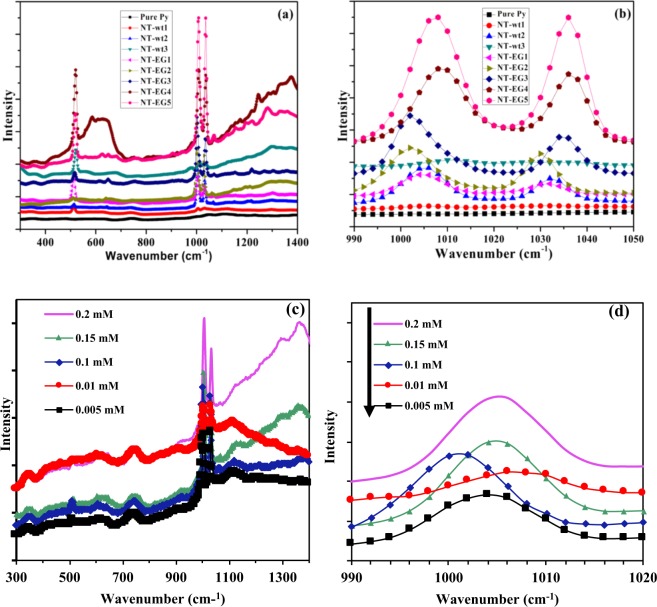
(**a**,**b**) Raman spectra of undiluted pure pyridine and fabricated AgNTs in different etchant solutions and concentration of AgNO_3_ fabricated on silicon substrate, and (**c**) and (**d**) Raman spectra of Py with different concentrations dripped on AgNTs-EG5.

Raman measurements were performed by using Micro Raman setup (ProRaman-L Analyzer PRO-L-5B1S) with an excitation laser beam wavelength of 532 nm with maximum power of 50 mW with an accumulation time was varied between 60 and 30 seconds for each set of experiments. Figure 4a,b, with an accumulation time of 60 seconds, shows the SERS spectra of Py with concentration of 0.2 mM for 8 different samples. The combination of Fig. 1 with Fig. 4b clearly shows the correspondence between the morphology of AgNTs, and the amplification of a Raman spectrum as expected. This fact can be explained on the basis of increasing the number of “hot spots”. In the SERS substrates fabricated by using EG, because of the better divergence of tree branches, the number of sharp ‘lighting’ tips as well as of ‘nano-gaps’ increased. Thus the number of ‘hot spot’ to amplify the Raman signal has become much greater. Consequently, the SERS enhancement of AgNTs-EG is significantly higher compared with AgNTs-wt^45^.

First peak appeared around 520 cm^*−*1^ in all 8 samples. The detection of Py in all AgNT-EG is shown clearly in Fig. 4b. Two strong Raman band at about ~ 1004 and 1032 cm^*−*1^ were clearly observed in the spectrum at 7 samples. Sample 1 that has the lowest concentration of silver and fabricated without EG did not have any peak of Py. The peaks of Py are enhanced with increasing the silver concentration with using EG in growth process. To find out the detection limit for Py of AgNTs-EG, we have used AgNTs-EG5 to record the Raman spectra of Py with different concentrations, including 0.005, 0.01, 0.1, 0.15 and 0.2 mM at peak ~ 1004 cm^*−*1^ at an accumulation time of 30 seconds. The signal intensity increased concomitantly with an increase in Py concentration as shown in Fig. 4c,d. We calculate the enhancement factor (EF) as the ratio between the intensity of the SERS to that of pure pyridine. The reason we used this way of calculation is that other techniques for the calculation of the EF assume nanoparticles (nano-spheres) with specific particle diameter which is not the case in the manuscript since we are studying nanotrees. Using this definition of the enhancement factor as follows:

$$EF=\frac{{I}_{sers}}{{I}_{pure}},$$

where I is the intensity at a certain wavenumber. Using this equation we have an enhancement factor of 16 at wavenumber 1008 cm^−1^ for AgNT-EG5 as shown in Fig. 4a. Although the EF reported in literature are in orders of powers of 10, however, those EFs do take into account the geometrical parameters of the sample as explained above. Otherwise, the ratio of the intensities would be in the range of 5 to 10 as reported in literature^46^. As shown in Fig. 4c,d, EF is around 2.5. However, such EF value can be enhanced for higher accumulation time.

## Experimental Method

The n-type (111) oriented mono-crystalline Si wafer with a resistivity of 0.002–0.0045 Ω cm was used to template the formation of Ag Nano-trees. The Si samples were cleaned as reported in our previous work. After the cleaning procedures, Si samples were divided to two groups, first group immersed in aqueous solution A that contained 10% HF and different concentrations of silver nitrate (5, 7.5, 10, 12.5, 15) mM for 1 minute with stirring at room temperature, to fabricate samples AgNT-wt1, AgNTs-wt2, AgNTs-wt3, AgNTs-wt4 and AgNTs-wt5, respectively. The second group of Si samples was immersed in aqueous solution B that contained 10% HF, 10% of ethylene glycol and different concentrations of silver nitrate (5, 7.5, 10, 12.5, and 15) mM for 1 minute also with stirring at room temperature, to fabricate samples AgNT-EG1, AgNT-EG2, AgNT-EG3, AgNT-EG4, and AgNT-EG5 respectively. The above steps were performed five times for all samples to make sure that the same results could be obtained every time with following the same steps. For SERS measurements, the pyridine with different concentrations was added drop wise on the substrate surface.

## Conclusions

The AgNTs deposited on silicon have been fabricated via one step electroless method in one minute. The density of AgNTs could be controlled by changing the concentration of AgNO_3_ through etching process. The absorption of AgNTs-EG is higher than the absorption of AgNT-wt in all concentrations because of higher refractive index of ethylene glycol. Moreover, AgNTs-EG have high enhancement factor of SERS. The AgNTs-EG can detect Py with concentration as low as 0.005 mM, while AgNTs-wt can only detect Py with concentration as low as 0.2 mM.
